# Characterization of Virulence Genotypes, Antimicrobial Resistance Patterns, and Biofilm Synthesis in *Salmonella* spp Isolated from Foodborne Outbreaks

**DOI:** 10.1155/2024/4805228

**Published:** 2024-09-20

**Authors:** Mohammad Mehdi Soltan Dallal, Ahmad Nasser, Samira Karimaei

**Affiliations:** ^1^ Food Microbiology Research Center Tehran University of Medical Sciences, Tehran, Iran; ^2^ Department of Pathobiology School of Public Health Tehran University of Medical Sciences, Tehran, Iran

## Abstract

*Salmonella* is the main bacterial pathogen that causes foodborne disease, particularly in developing countries. Nontyphoidal *Salmonella* (NTS) include *Enteritidis* and *Typhimurium* as the most prevalent strains which are one of the significant causes of acute gastroenteritis in children. Therefore, identifying the most predominant serovars, types of common contaminated food, and paying attention to their antibiotic resistance are the main factors in the prevention and control strategy of salmonellosis. This study was undertaken to evaluate the prevalence rate of serovars, the biofilm formation, antimicrobial resistance (AMR) status, and phenotypic virulence factors of *Salmonella* strains isolated from diarrhea samples in some cities of Iran. A total of 40 (10.41%) *Salmonella* isolates were recovered from 384 diarrhea samples processed and the most common serovar was *Salmonella* serovar *Typhimurium* (82.5). Also, all isolates belonging to serovar *Typhimurium* showed more virulence factors compared to other serovars. The isolates showed a high resistance rate to ampicillin (95%) and nalidixic acid (87.5%), while a low resistance rate was found for chloramphenicol (2.5%). Moreover, significant variances in the capacity of biofilm formation were found between different *Salmonella* serotypes. The resistance of NTS to extant choice drugs is a potential public health problem. Constant monitoring of AMR pattern and virulence profile of NTS serovars is suggested for the prevention of salmonellosis in humans.

## 1. Introduction

*Salmonella enterica* serovars is the main cause of foodborne outbreaks which has led to global public health concerns [1]. This bacterium is annually involved in approximately 94 million cases of salmonellosis and 155,000 deaths worldwide [2]. In terms of epidemiology, a variety of food products have been described as the major source of *Salmonella* infections. Nontyphoidal *Salmonella* (NTS) is the main zoonotic pathogen described in children, transmitted through contaminated food or water ingestion or exposure to polluted animals or poultry [3, 4]. In addition, *Salmonella enterica* serovar *Typhimurium* and *S. Enteritidis* are the most predominant serotypes related to NTS gastroenteritis in children worldwide which is usually self-limiting [4]. NTS gastroenteritis is described with abdominal cramps, fever, nausea, and vomiting, where hospitalization may be essential for severe cases including susceptible and immunocompromised individuals (such as children and HIV-positive, or diabetics cases) [5].

Invasive NTS infections are life-threatening infections particularly for infants and young children, as well as those stressed with malnutrition, the elderly, and immunocompromised individuals [6]. The 2017 Global Burden of Diseases, Injuries, and Risk Factors Study (GBD) assessed that *Salmonella enterocolitis* causes approximately 95.1 million cases, 50771 deaths, and 3.10 million DALYs [7]. In addition to causing diarrheal disease, nontyphoidal *Salmonella* infections can cause bacteremia, meningitis, and other focal infections by invading normally sterile sites [8]. These infections, referred to as invasive nontyphoidal *Salmonella* disease, usually do not cause diarrhea, but present as nonspecific febrile illnesses with clinical symptoms similar to and indistinguishable from other febrile illnesses, and with higher mortality cases than is seen in noninvasive infections [8].

The presence of *Salmonella* pathogenicity island (SPI) can be characterized as a benefit for bacteria that cause the infection. *Salmonella* pathogenicity island 1 (SPI1) exists in approximately all *Salmonella*, which is a genetic element on the chromosome comprising virulence genes, which encode factors involved in invasion [3]. Moreover, SopB is an effector protein that is involved in prompting fluid secretion, encoded by *Salmonella* pathogenicity island 5 (SPI5) [9]. *Salmonella* invades host cells and survives in the Peyer's patches via the expression of two genes, *sopE* and *gipA*, which are transferred through phages [10, 11]. Another phage-related gene is *sodCI* which preserves *Salmonella* against oxidative degradation [12]. *Salmonella* pathogenicity island 2 (SPI2) is a preserved chromosomic element that permits it to endure the conditions inside the macrophage and spread in the host body [3]. Finally, *Salmonella* pathogenicity island 3 (SPI3) and SPI5 encode some effector factors that alter the macrophage conditions [3].

The widespread use of antimicrobial agents in clinics and farming has caused the emergence of antimicrobial resistance (AMR) in NTS, which is a global concern [13, 14]. Moreover, the increase of multidrug resistance (MDR) has been reported for *Salmonella* obtained from food products [15]. Hence, high antibiotic resistance has been reported for *Salmonella*, especially to ampicillin, sulfonamides/sulfamethoxazole, and tetracycline in Europe [16]. In addition, the development of quinolone resistance is the main concern for the effective control and management of salmonellosis [17]. Antibiotic resistance genes can be carried on mobile elements such as plasmids and integrons [18]. In particular, several antibiotic resistance cassettes, carrying diverse combinations of resistance genes, were described in *Salmonella* integrons [19]. Meanwhile, the level of resistance in NTS differs with various serotypes and in diverse geographical regions, knowledge of common serovars and their AMR patterns is valuable in developing empirical treatment guidelines and monitoring the spread of this pathogen in the community [4, 20]. Recently, a new type of multidrug-resistant *Salmonella Typhimurium*, ST 34, was described in Vietnam, which was associated with invasive disease in immunocompromised patients [21]. Antimicrobial-resistant infections cause poorer clinical results and higher mortality rates [22].

The ability of bacteria to form biofilms as well as the growing problem of antimicrobial resistance (AMR) is the main cause of therapeutic problems in humans [23, 24]. The biofilm formation ability is variable in *Salmonella* and influenced by various sources or serotypes of the isolates [25]. *Salmonella enterica* serovars include *Salmonella Enteritidis* and *Salmonella Typhimurium,* which cause the most cases of salmonellosis with a noticeable enhancement in the biofilm formation ability [25].

This study aims to characterize some virulence traits in *Salmonella* spp. isolates from humans. Particularly, the isolates have been assessed for (i) the presence of several virulence genes: *invA*, *spvB*, *prgH*, *sopB*, *mgtC*, *pipB*, *rhuM*, and *gipA*; (ii) resistance profiles against six antibiotics; and (iii) the ability to produce biofilm.

## 2. Materials and Methods

### 2.1. Sample Collection, Bacterial Isolation, and Characterization

Annually, numerous foodborne diseases are reported by NTS strains in Iran. Hence, it is worth noting that in the period between 2013 and 2019, among the 2098 cases reported about foodborne outbreaks in Iran, 350 cases (16.7%) were associated with *Salmonella* [26]. From September 2021 to June 2022, 384 diarrhea samples were collected from patients afflicted with gastroenteritis-detected *Salmonella* spp. The age range of the patients was between 3 and 70 years old, moreover, 216 (56.25) and 168 (43.75) of them were males and females, respectively. Sampling was performed on patients who had watery, loose, or bloody stools more than 3 times per day. Isolates were detected as *Salmonella* using conventional biochemical and serological assays. In brief, the stool samples were transported into selenite-F broth and within 4 h at 4°C to the bacteriology laboratory of the department of pathobiology, Tehran University of Medical Sciences. In the following, subculturing was performed on MacConkey agar and suspected colonies were identified by standard biochemical assays including oxidase, methyl red-Voges–Proskauer (MRVP), catalase, citrate consumption, TSI agar, and urease production [27].

### 2.2. Serotyping of Positive Samples

The slide agglutination assay, using Difco antisera, was performed for *Salmonella* serotyping and then interpreted based on the Popoff method [28]. After confirming the isolates as *Salmonella* by biochemical tests, serotyping with antisera O (B, D, E, and C) and H was performed to characterize O and H antigens.

### 2.3. Molecular Detection of Virulence Genes

*Salmonella* isolates were subjected to DNA extraction by the boiling method as described previously [27] and to a panel of PCR assay to assess the presence of some virulence-associated genes: *invA*, *spvB*, *prgH*, *sopB*, *mgtC*, *pipB*, *rhuM*, and *gipA*. Primers used for the present study are described in Table 1. The PCR assay was performed in a final volume of 20 *μ*l containing 10 *μ*l PCR Master Mix (SinaClon Bioscience, Iran), 1 *μ*l of forward and reverse primers, and 1 *μ*l of template DNA. The PCR reaction conditions are shown in Table 1. The amplification products were subjected to electrophoresis in a 1.5% agarose gel with the 100 bp DNA Ladder (Fermentas, Lithuania) and stained with GelRed™ (Biotium).

### 2.4. Antimicrobial Susceptibility Test

The antibiotics were selected according to their common use in the prevention and treatment of *Salmonella* infections in humans. Susceptibility to antimicrobials was determined with the disc diffusion test according to the Clinical and Laboratory Standards Institute's (CLSI) manual [33]. Eleven antibiotics were examined including ampicillin (AMP), tetracycline (TE), ceftriaxone (CRO), ciprofloxacin (CIP), imipenem (IPM), gentamycin (GEN), cefotaxime (CTX), cefoxitin (FOX), nalidixic acid (NA), chloramphenicol (CHL), and trimethoprim/sulfamethoxazole (SXT). The resistance profiles were categorized into three following patterns: MDR, XDR, and PDR which were established by Magiorakos et al. [34]. *Escherichia coli* ATCC 25922, which is susceptible to all of the antimicrobials, was used as quality control.

### 2.5. Biofilm Formation Assay

To evaluate the formation of bacterial biofilm, the crystal violet protocol was applied as previously described [35]. After conventional biochemical confirmation, a single *Salmonella* colony was selected and inoculated in trypticase soy broth (TSB, Merck, Germany). After overnight incubation at 37°C, *Salmonella* cultures were diluted 1 : 10 in TSB with 0.5% glucose.

Next, 100 *μ*l of this diluted bacterial culture was transferred to a 96-well, flat-bottomed microtiter plate. The plates were then placed at 37°C for 48 h and washed three times with 200 *μ*l of sterile PBS 1X to discard nonadherent bacterial cells. Crystal Violet stain (1%) was added to each well and incubated at room temperature for 15 min. Each well was then washed with PBS three times as before to remove the extra dye. Finally, 100 *μ*L of 95% ethanol was added to dissolve crystal violet dye bound to the adherent cells and the plates were evaluated at 620 nm with a spectrophotometer. The cut-off value (ODc) is specified as three standard deviations (SDs) above the mean OD of the negative controls. The isolates were classified into the following four groups based on the ODs obtained: OD ≤ ODc: nonadherent; ODc < OD ≤ 2 × ODc: weak biofilm producer; 2 × ODc < OD ≤ 4 × ODc, moderate biofilm producer; and 4 × ODc < OD, strong biofilm producer. It is worth mentioning that a well with only medium and one with *Pseudomonas aeruginosa* PAO1 were considered as negative and positive controls, respectively. Also, the assay for each isolate was performed three times.

### 2.6. Statistical Analysis

The results of biofilm formation were presented as the mean ± standard deviation. One-way analysis of variance (ANOVA) was applied to assess the differences in biofilm formation capacity between the different *Salmonella* serotypes by GraphPad Prism software 8 (GraphPad Software Inc.). The level of significance was *P* value < 0.05.

## 3. Result

### 3.1. Prevalence and Identification of *Salmonella* Isolates

Overall, 112 *Salmonella-*associated foodborne outbreaks occurred between 2021 and 2022 in Iran. Out of the 384 diarrhea samples examined, 40 (10.41%) *Salmonella* isolates were recovered. As shown in Table 2, the details such as the age and gender of each patient from whom *Salmonella* was isolated were recorded.

### 3.2. *Salmonella* Serovar Identification

Among the forty *Salmonella* isolates, four different serovars were identified including *Typhimurium, Infantis, Paratyphi B, and Choleraesuis.* Moreover, S. ser. *Typhimurium* was the most detected serovar with 33 isolates, and two isolates were detected for each of the following serovars: *Paratyphi B and Infantis*. Only one isolate belonged to S. s*er. Choleraesuis.* Finally, further two isolates were found belonging to the genus *Salmonella*, but they were not typable.

### 3.3. The Molecular Identification of Virulence Genes

Virulence-associated genes were detected in all 40 studied isolates, consistent with what was expected for pathogenic *S. enterica* strains. In addition to the identification of the *Salmonella* genus, the *invA* gene is involved in enterocyte invasion and is a virulence-related gene [13] that was found in all investigated isolates. Regarding the presence of virulence-related genes, the most detected genes were *mgtC*, *pipB*, and *sopB*, with 34/40 (85%), 36/40 (90%), and 38/40 (93%) positive isolates, respectively. As shown in Table 3, two genes *rhuM* and *spvB* were detected in less than approximately 70% of the isolates, while the *gipA* gene was identified only in one isolate and none of the *Salmonella* isolates had the *prgH* gene. The detection of *sopB*, *mgtC*, *rhuM*, *pipB*, and *spvB* genes along with *invA* was detected in *S. Typhimurium* isolates. The majority of isolates (33/40, 82.5%) could amplify these gene fragments (Table 4). Only one isolate was confirmed as *S. choleraesuis* by identifying genes *sopB* and *gipA*. In the case of two strains (5%), genes *sopB* and *pipB* were detected and confirmed as *S. infantis*. Three genes *sopB*, *pipB*, and mgtC resulted in association to *S. paratyphi* in two isolates. Finally, regarding genetic characteristics, two isolates were classified as *Salmonella* spp due to only the invA gene being detected in them.

### 3.4. Antimicrobial Resistance Profiles

All *Salmonella* isolates (100%) were susceptible to imipenem, and only one isolate was resistant to chloramphenicol. The *Salmonella* isolates showed a high antimicrobial resistance rate to some tested antibiotics, so 38 isolates were resistant to ampicillin (95%). Also, a high percentage of isolates (87.5%) resulted in resistance to nalidixic acid. The percentage of resistance to used antibiotics is shown in Table 5. MDR was found in 18/40 (45%) of the studied isolates. A detailed consideration of the *Typhimu*rium serotype and comparison with other serotypes resulted in the association of MDR to these serotypes with a statistically significant difference (*p* ≤ 0.05). The most common MDR profile was AMP FOX NA SXT and included ten isolates with nine isolates belonging to the *Typhimurium* serotype and one to the *Infantis* serotype. The CRO AMP GEN SXT profile was characteristic of the two isolates belonging to the *Typhimurium* serotype, and the AMP GEN NA SXT profile for the four isolates was also obtained from this serotype. The AMP TE NA SXT profile involved two isolates from *Typhimurium* (one isolate) and *Paratyphi B* (one isolate).

### 3.5. Biofilm Formation

The microtiter plate method was used to evaluate the biofilm formation capacity of *Salmonella* isolates. All 40 tested *Salmonella* isolates could form biofilm on polystyrene surfaces after 48 h. The mean OD value for all 40 *Salmonella* isolates was 0.6 ± 0.02, whereas 32.5% of all isolates were categorized as strong biofilm producers. Moreover, the results showed that 15 (37.5%) isolates were weak biofilm producers while 12 isolates (30%) had moderate capacity for biofilm formation. Significant variances in the capacity to produce biofilm were found between different *Salmonella* serotypes (*p* < 0.05) (Table 6). S. ser. *Infantis* (OD620 nm = 0.36 ± 0.01) and S. ser. *Paratyphi B* (OD620 nm = 0.48 ± 0.12) were all categorized as moderate biofilm producers. The capacity of S. ser. *Choleraesuis* and 11 isolates of S. ser. Typhimurium to produce biofilm was significantly higher than other serotypes, so classified as a strong biofilm producer. Also, among 33 isolates of S. ser. *Typhimurium*, 7 (17.5%) were moderate biofilm producers and 15 (37.5%) were weak biofilm producers. Regarding two isolates that could not be typeable by serotyping, it was determined that one strain could produce strong biofilm (OD620 = 1.24 ± 0.01) and the other strain could produce moderate biofilm (OD620 = 0.56 ± 0.01).

## 4. Discussion

Despite public health promotion, the CDC reported that the Salmonellosis prevalence and the morbidity rate subsequent to its infections are also still high [36]. *Salmonella* remains the second most common foodborne pathogen in the United States and Europe [37, 38]. Its infections are self-limiting with no need for antibiotic treatment except in antibiotic-resistant cases that have affected public health [39]. Therefore, continuous monitoring of *Salmonella* isolates is necessary not only to investigate the prevalence but also to consider their virulence profile.

The WHO Global Network of Foodborne Infections about NTS surveillance has reported that *S. Enteritidis* and *S. Typhimurium* are the prevalent serovars obtained from humans in both developed and developing countries [4]. The presence of animal reservoirs or local food plays an important role in the dominance of certain serotypes in some geographical areas. Remarkably, *Salmonella* serotype distribution in a certain region may differ over a period of time [4]. Hence, the results indicated that the most common serovars were S. ser. *Typhimurium* (33/40, 82.5%) which was involved in *Salmonella* outbreaks in Iran. While, our group previously reported that two serovars S. ser. *Enteritidis* and S. ser. *Senftenberg* were the most recorded serovars in Iran, particularly in foodborne outbreaks [5]. Similar to the results of the present study, among 990 *Salmonella* foodborne outbreaks that occurred in Australia, S. ser. *Typhimurium* with a frequency of 84% was the most prevalent serovar [40]. However, another study in the United States indicated that S. ser. *Enteritidis* (24.4%) was the most prevalent serovar identified from foodborne outbreaks between 2009 and 2014 [41].

Regarding pathogenic features, the presence of several genes related to virulence, located on SPI3 and SPI5, prophages/plasmids, was considered. It is broadly confirmed that the presence of these genes or the induction of their expression confers some advantages for bacteria [29]. The *invA* gene was detected in all *Salmonella* isolates which is associated with host recognition and invasion of intestinal cells [29]. This gene is located on *Salmonella* pathogenicity islands and is related to the structure of the type three secretion system (TTSS III) and is the key target gene for the recognition of strains belonging to the genus *Salmonella* [29]. The *invA* gene detection in all the analyzed isolates is in agreement with former studies worldwide [29, 42, 43]. A percentage of 95% of the studied strains possessed the *sopB* gene. This gene is located on SPI5 and participates in invasion and prompting fluid secretion and it is often found in *Salmonella* clinical isolates [44]. Another gene located in SPI5 is *pipB*, which is involved in bacterial survival in the host cell and systemic dissemination [45]. The *pipB* gene was detected in 36 (90%) analyzed isolates. Another most identified gene was *mgtC* (34/40, 85%) which is usually detected in clinical isolates. The *mgtC* gene is located in SPI3 and allows the bacterium to enter the phosphate into the bacterial cell within the host's macrophage [46]. This protein can target the histidine-kinase PhoR system independently of the presence of phosphate and deletion of this gene disrupts phosphate absorption and inhibits hypervirulent *Salmonella* [46]. The *gipA* genes may be carried by phages, involved in M cell invasion in the Peyer's patches, which will meaningfully enhance *Salmonella* toxicity. This gene is not commonly detected in *Salmonella* strains. Similarly, in the present study, the presence of this gene was positive only in one isolate (2%). The *rhuM* gene located on SPI3 that participated in systemic dissemination was found in 27 isolates (67%). Finally, 24 isolates had the *spvB* gene (60%) which has plasmid origin and is related to the *Salmonella* virulence plasmid and involved in the bacterial maintenance and survival inside the host cell [29].

Based on the results, most isolates had at least three virulence genes *invA*, *sopB*, and *pipB* which the high frequency of these virulence genes indicates their ubiquity and conserved structure. Previously, it was confirmed that the three following genes: *invA*, *mgtC*, and *sopB* are highly conserved and used as genetic markers for the detection of *Salmonella* pathogenicity island [47]. Our results proposed that more virulence-related genes are often related to the serotype *Typhimurium*, which shows more pathogenicity and virulence of *Salmonella* belonging to this serovar. Moreover, other serotypes had a low frequency in our study and the rare reports about other serovars in humans can reflect their low pathogenicity. In fact, without a control system, only clinical isolates are identified that are associated with human infections and require care and hospitalization, while infections may be caused by low-frequency strains that have not resulted in clinical disease.

Antibiotic resistance is a concerning public health problem in *Salmonella*. The results indicated that all isolates were susceptible to imipenem, while there was a high percentage of resistance (≥80%) to some tested antibacterial drugs (Table 5). Although carbapenem resistance is increasingly reported in *Enterobacteriaceae*, it is still very rare in *Salmonella* [48]. Carbapenems are usually used as the last choice for the treatment of MDR Gram-negative bacterial infections [48]. *Salmonella* strains are usually susceptible to carbapenems and this antibiotic is used as the last choice in salmonellosis treatment [48]. The susceptibility of all isolates to imipenem in the present study can be an indicator for public health in the treatment of *Salmonella* infections that this antibiotic is still effective. The third/fourth-generation cephalosporins are mostly recognized as crucial antimicrobials for the treatment of *Salmonella* severe infections [49]. In the present study, only two isolates (5%) were resistant to ceftriaxone. In another study, the amount of resistance to ciprofloxacin in Salmonella isolates was reported to be negligible, in accordance with our results [50]. Although aminoglycosides are commonly administered as growth promoters in animals, these agents are not recommended for the treatment of salmonellosis [51]. The results of the present work showed that 22.5% of *Salmonella* isolates were resistant to gentamicin. In contrast to our findings, other surveys showed a much higher resistance to aminoglycoside [52, 53].

The results displayed a high-level MDR (45%), which was similar to a previous study by Nguyen Thi et al. [3]. The simultaneous resistance to cefoxitin, ampicillin, nalidixic acid, and trimethoprim/sulfamethoxazole was the most common pattern, presented by ten isolates belonging to serovars *Typhimurium (*nine isolates) and *Infantis* (one isolate). Therefore, evaluation of antimicrobial resistance confirmed that resistant strains and MDR patterns were more associated with serotypes Typhimurium. These results may help to explain why infection by these serotypes requires specific treatment and occasionally hospitalization, and it confirms that they can be more dangerous for human health.

*Salmonella* as well as other *Enterobacteriaceae* species can adhere and form biofilm on the surface of various materials during their life cycle [54]. Biofilms may be critical for the survival of *Salmonella* in adverse conditions [23]. The data of the present study displayed that the ability of biofilm formation was affected by the serotype of *Salmonella* strains, which is consistent with previous reports [25, 55].

## 5. Conclusions

The results showed great variability among *Salmonella* serovars and strains circulating in humans. Although ranking is not possible, some serovars were more virulent than others, S. ser. *Typhimurium* particularly displayed several studied pathogenic characteristics and this evidence verified *Typhimurium* as the most important virulent serovar. In the present study, resistance to fluoroquinolones and ciprofloxacin was observed in some NTS serovars, which can cause concern and threat to public health. Unnecessary use of antibiotics for treating NTS infection should be limited. On the other hand, the isolation of MDR *Salmonella* isolates is worrying and supports the importance of monitoring *Salmonella* serovars and their AMR pattern to prevent the spread of resistant isolates.

## Figures and Tables

**Table 1:** Primers used for detection of virulence genes in *Salmonella* isolates.

Genes	Sequence 5′ ⟶ 3′	Amplicon size (bp)	Tm	References
*spvB*	F: CTATCAGCCCCGCACGGAGAGCAGTTTTTA R: GGAGGAGGCGGTGGCGGTGGCATCATA	717	56	[29]

*invA*	F: CTGGCGGTGGGTTTTGTTGTCTTCTCTATT R: AGTTTCTCCCCCTCTTCATGCGTTACCC	1070	53	[29]

*prgH*	F: GCCCGAGCAGCCTGAGAAGTTAGAAA R: TGAAATGAGCGCCCCTTGAGCCAGTC	756	66	[29]

*sopB*	F: CGGACCGGCCAGCAACAAAACAAGAAGAAG R: TAGTGATGCCCGTTATGCGTGAGTGTATT	220	53	[29]

*mgtC*	F: GGCAGGAGTTTCGCACTAAC R: GCGTACCCACAATGGATTTC	444	59	[30]

*pipB*	F: AATATCGGATGGGGGAAAAG R: AACCTGACTCACGCAGACCT	230	55	[31]

*rhuM*	F: CATCGGCTGTACCCGACTAT R: CAGCACGCTGATGAATGAGT	222	55	[31]

*gipA*	F: ACGACTGAGCAGGCTGAG R: TTGGAAATGGTGACGGTAGAC	422	59	[32]

Primary denaturation	Denaturation	Annealing	Extension	No. of cycles

94°C 5 min	94°C 30 s	59°C 30 s	72°C 60 s	35

**Table 2:** Demographic status (age and gender distribution) of the patients along with the city of isolation for each *Salmonella* isolate.

*Salmonella* isolates		Patients	
ID	City	Gender	Age
K1	Karaj	Female	21
K2	Karaj	Male	19
K3	Karaj	Male	45
K4	Karaj	Female	13
K5	Karaj	Male	54
K6	Karaj	Male	60
K7	Karaj	Male	7
K8	Karaj	Female	5
K9	Karaj	Male	68
K10	Karaj	Male	4
K11	Karaj	Male	49
K12	Karaj	Male	25
K13	Karaj	Male	10
K14	Karaj	Female	55
K15	Karaj	Male	14
T1	Tehran	Male	66
T2	Tehran	Male	65
T3	Tehran	Female	40
T4	Tehran	Male	38
T5	Tehran	Female	18
T6	Tehran	Male	6
T7	Tehran	Female	17
T8	Tehran	Male	36
T9	Tehran	Female	7
T10	Tehran	Male	20
T11	Tehran	Male	42
T12	Tehran	Female	5
T13	Tehran	Male	43
T14	Tehran	Male	59
S1	Semnan	Male	61
S2	Semnan	Female	62
S3	Semnan	Male	3
S4	Semnan	Male	21
S5	Semnan	Female	12
S6	Semnan	Male	4
S7	Semnan	Male	63
S8	Semnan	Male	10
S9	Semnan	Female	56
S10	Semnan	Male	18
S11	Semnan	Female	16

**Table 3:** Frequencies of virulence genes screened by PCR assay in *Salmonella* isolates.

Gene function	Gene	Absolute frequencies (*n* = 40)	Relative frequencies (%)
Growth within host	spvB	24	60
Host recognition/invasion	invA	40	100
Host recognition/invasion	prgH	0	0
Host recognition/invasion	sopB	38	95
Survival within macrophage	mgtC	34	85
Survival within host cells	pipB	36	90
Involved in systemic dissemination	rhuM	27	67
Survival in Peyer's patches	gipA	1	2

**Table 4:** Virulence genes' profile of *Salmonella* isolates.

Virulence profile	Serotype	Virulence genes	*N* (%)
VP1		*invA*	2
VP2	*Typhimurium*	*invA-sopB-mgtC-rhuM-pipB-*spvRBC	33
VP3	*Choleraesuis*	*InvA-sopB-gipA*	1
VP4	*Infantis*	*InvA-sopB-pipB*	2
VP5	*Paratyphi*	*InvA-sopB-pipB-*mgtC	2

**Table 5:** Antimicrobial resistance of *Salmonella* spp. isolates.

Antimicrobial	Susceptible	Intermediate	Resistant	Nonsusceptible	%
	No	No	No	No	
SXT	19	0	21	21	52.5
CIP	37	0	3	3	7.5
IPM	40	0	0	0	0
CHL	39	0	1	1	2.5
AMP	2	14	24	38	95
TE	38	0	2	2	5
GEN	31	2	7	9	22.5
CTX	37	1	2	3	7.5
CRO	38	0	2	2	5
FOX	26	4	10	14	35
NA	5	11	24	35	87.5

Antimicrobials: SXT, trimethoprim/sulfamethoxazole; CIP, ciprofloxacin; IMI, imipenem; CHL, chloramphenicol; AMP, ampicillin; TE, tetracycline; GEN, gentamicin; CTX, cefotaxime; CRO, ceftriaxone; FOX, cefoxitin; NA, nalidixic acid.

**Table 6:** Biofilm formation capacity of different *Salmonella* serotypes.

Biofilm formation						
*Salmonella* serotypes	Weak biofilm producer		Moderate biofilm producer		Strong biofilm producer	
	No. (%)	Average OD ± SD	No. (%)	Average OD ± SD	No. (%)	Average OD ± SD
*Salmonella Typhimurium* (*N* = 33)	15 (37.5)	0.18 ± 0.01	7 (17.5)	0.44 ± 0.01	11 (27.5)	0.96 ± 0.01
*Salmonella Choleraesuis* (*N* = 1)					1 (100)	1.26 ± 0.08
*Salmonella Infantis* (*N* = 2)			2 (5)	0.36 ± 0.01		
*Salmonella Paratyphi B* (*N* = 2)			2 (5)	0.48 ± 0.12		
*Salmonella* spp			1 (2.5)	0.56 ± 0.01	1 (2.5)	1.24 ± 0.01
Total	15 (37.5%)	0.18 ± 0.01	12 (30%)	0.46 ± 0.03	13 (32.5%)	1.15 ± 0.03

OD, optical density; SD, standard deviation.

## Data Availability

The data used to support the findings of this study are available from the corresponding author upon reasonable request.
